# The affective component of learning in simulation-based education – facilitators’ strategies to establish psychological safety and accommodate nursing students’ emotions

**DOI:** 10.1186/s12912-022-00869-3

**Published:** 2022-04-20

**Authors:** Anine Madsgaard, Kari Røykenes, Hilde Smith-Strøm, Monika Kvernenes

**Affiliations:** 1 VID Specialized University, Ulriksdalen 10, 5009 Bergen, Norway; 2 University of Bergen, VID Specialized University, Bergen, Norway

**Keywords:** Emotions, Simulation training, Teaching, Nurse education

## Abstract

**Background:**

Active learning situations such as simulation-based education (SBE) are found to trigger a wide range of emotions among students. Facilitators have an important educational role in SBE which include being attentive and adaptive to students’cognitive and affective responses. Although the importance of emotions in SBE is recognized in facilitator guidelines, little is known about how facilitators accommodate student affect. Hence, this study explores facilitators’ strategies for addressing students’ emotions in SBE.

**Method:**

Individual interviews with nine facilitators were performed and transcripts were subjected to qualitative analyses in accordance with interpretive description approach.

**Results:**

Findings show that facilitators are attentive to and continuously assess students’ emotional responses in SBE. Both positive emotions, such as interest and surprise, and negative emotions such as anxiety are cultivated, yet adapted to the perceived needs of the individual student. Psychological safety was seen as a prerequisite for optimal learning, regardless of the students’ previous level of knowledge. Furthermore, significant learning was seen as something that might also arise from uncomfortable experiences, such as students realizing their own mistakes or uncertainty. Hence facilitators were found to balance levels of difficulty, emotional arousal and psychological safety during the various phases of SBE.

**Conclusion:**

Facilitators recognize the emotional dimension of learning in SBE and have numerous strategies for accommodating students’ emotions. This study highlights the complexity of the facilitator’s role in adapting training to individual cognitive and emotional needs. These findings have implications for facilitator training which should include awareness of the role of emotions in learning and strategies for observing and accommodating training to meet emotional needs.

## Background

In simulation-based education (SBE), the facilitator plays an essential role by creating student-centred learning environments to evoke student engagement and facilitate reflections [1]. Facilitators pedagogical responsibility is complex as they are expected to adhere to numerous SBE frameworks and guidelines, continuously monitoring learner progression and meeting high and complex expectations from students [1, 2].

During SBE, health professional students are challenged to handle practical clinical problems in simulated yet realistic contexts. According to Jeffries (2021), SBE is an experiential learning method consisting of three sequences: Prebriefing, scenario and debriefing. In prebriefing*,* students are introduced to the scenario and given the opportunity to familiarize themselves with equipment. Students then engage in scenario*,* where they act as health professionals in collaboration with peers and simulated patients. Learning is further enhanced during debriefing where students are encouraged to reflect on their own actions and those of others. SBE is performed in a realistic clinical environment and can be performed using technology with complex manikins, or using humans in roles as patients [1]. SBE has been found to be important for student learning of psychomotor skills, clinical knowledge, confidence and self-efficacy [3, 4].

SBE builds on experiential learning theory founded in a holistic perspective on learning [5]. Kolb describes learning as a continuous process grounded in experience followed by perception, cognition and change in behaviour. Applying Kolbs experiential model to SBE, the scenario is where the students engage in an experience, while perception and cognitions are found to happen during reflection in debriefing when students are given the opportunity to reflect upon thoughts, emotions and behavioural actions [1]. Learning occurs when students are actively engaged in an interplay between emotions, cognition and experiences [6]. Such bodily activation attaches itself deeper than cognitive surface learning [7]. Active learning situations such as SBE have been found to evoke students’ emotions [8].

The function and importance of emotions as part of student learning has been increasingly recognized over the past decades [9]. Emotions are researched from various perspectives, from neurobiological and physiological research to constructivist and phycological research. Emotions have been broadly defined as "*multifaceted phenomena involving sets of psychological processes, including affective, cognitive, physiological, motivational, and expressive components* [8]. In this study we use the term emotions in line with a constructivist approach to learning, seeing emotions as constructed experiences that are cognitive episodes evoked by stimuli. Emotions are short-lived and are characterized by subjective feelings, physiological changes, tendency to act and an appraisal component. For instance, in the appraisal of a dangerous situation, one will feel fear, changes in pulse and blood flow followed by the act of running away. Affect refers to a person’s immediate emotional reaction to situations and is associated with the subjective experiential component in an emotion [10].

In the context of higher education, emotions are particularly relevant because they have been found to influence students’ motivation, perceptions, cognition and memory and are therefore significant to both the learning process and its outcome [11, 12]. Emotions can be helpful or unhelpful during educational activities [13]. A traditional understanding of emotions’ impact on learning has been that negative emotions e.g. anxiety, hinder learning while positive emotions, e.g. interest, motivate learning. However, research shows that what we commonly view as negative emotions can in fact motivate learning, increase attention and stimulate cognition [14, 15]. At the same time, positive emotions can cause unproductive relaxation [16]. Nursing students’ anxiety and stress in SBE have been described through numerous studies [17–19], and the importance of psychological safety has been highlighted. Furthermore, students’ emotions are seldom stable or unidimensional during academic tasks which should be considered when teaching [20]. Students participating in SBE were found to have emotional profiles spanning from engaged to neutral and anxious [21]. Emotional regulation has been put forward as key to turn emotionally charged experiences into optimal learning situations. Since emotions are reactions to specific situations, it is possible to regulate the learning situation in order to elicit specific emotions that can be beneficial for learning [22]. Hence, educators can improve learning outcomes, for instance by attempting to reduce fear when establishing a psychologically safe learning environment.

Although it must be assumed that competent facilitators recognize students’ emotions and regulate students’ emotions to elicit helpful emotions in educational setting, little is known about how facilitators approach the emotional aspect of learning in SBE.

### The facilitator’s role in SBE

Facilitators guide students through all three phases of SBE. Their role includes planning and conducting the simulation, preparing equipment, establishing a pedagogical learning environment, creating scenarios and facilitating reflection in the debriefing to ensure that learning objectives are achieved [23, 24].

To fulfil these multiple roles, facilitators need a broad set of competencies. Facilitators should understand the pedagogical principles that underpin simulation as a teaching strategy, be familiar with students’ previous knowledge, identify students’ knowledge gaps, have feedback competency and have the skills to elicit students’ reflections. Providing non-threatening feedback during debriefing is considered one of the most challenging tasks [25]. Further, facilitators should be able to recognize moments during scenario that are suitable for reflecting on in the debrief. Finally, facilitators should make it clear to students how knowledge gained in SBE is transferable to future practical settings [23, 26–28]. SBE guidelines highlight facilitators’ abilities to communicate clearly, create safe learning environments, maintain fidelity, regulate their teaching methods to fit students’ expertise and knowledge, continuously evaluate student knowledge and behaviour, and generally support learning throughout the SBE sequences [3, 23, 26, 27]. On a more personal level, excellent facilitators are described as being flexible, positive, enthusiastic, motivational, well prepared, and trustworthy [24, 26, 29]. In addition to managing the SBE method, facilitating also includes demonstrating up-to-date clinical skills and the need to be abreast of the current national standards. Clearly, the facilitator’s role is associated with high and varied expectations. The overall intention is to guide students to meet the described learning objectives. Various frameworks exist to help facilitators navigate these expectations. Promoting Excellence and Reflective Learning in Simulation (PEARLS) is one example, a framework used as a checklist for facilitators when debriefing in educational settings. PEARLS incorporates debriefing best practice through 28 essential points, including acknowledging students’ emotional reactions, focusing on promoting students’ self-assessment and providing feedback [30].

Previous research shows that facilitators’ conceptions of teaching in SBE are mainly focused on the transmission of knowledge and skills [31]. Further, safe learning environments were found to be an essential part of facilitating and are successfully implemented when students are treated individually. Harder (2010) described numerous methods facilitators use to improve SBE, including preparing students by clarifying expectations, challenging students by increasing the complexity of the scenario and not stopping scenarios that did not run well [32]. Facilitating was also found to include understanding when students need assistance and assessing when students are ready to continue the task [33]. Competent facilitators organized SBE, established and managed high clinical realism and performed SBE with professional values and identity [34]. 

### Psychological safety and risk-taking

Although SBE is popular among students, it is also associated with pressure to perform in front of peers, which is associated with stress and performance anxiety. Establishing a psychologically safe learning environment is essential, and particularly when the scenario is complicated, demands interactions or consists of multiple uncertain manoeuvres [24, 35]. Psychological safety is fundamental to students’ learning when they are exposed to such risks.

The learning environment is considered psychologically safe when students subjectively experience the situation as positive, non-threatening and non-judgemental. It is characterized by an atmosphere where they feel they can be themselves, where they trust each other’s intentions, wish each other well and where there is room for error without fear of negative consequences [29, 36]. SBE frameworks and guidelines highlight the facilitator’s responsibility to reduce student anxiety and establish psychological safety [1, 37–41], and facilitators are given plentiful advice on how to achieve this. Psychological safety is found to be established through comprehensive group dynamics and interpersonal relationships, when facilitators recognize input and ideas, encourage students to ask questions and destigmatize failures [36, 42]. The importance of students having the opportunity to become familiar with the equipment, monitors and manikins are highlighted as stress-reducing moves [2, 43]. It is suggested that facilitation can be displayed by preparing well-organized simulations, demonstrating trustworthiness and accessibility, and being supportive and open [29, 36, 44, 45]. Facilitators’ perceptions of playing a role in establishing psychological safety were identified in Kostovich et al.’s study, and facilitators were found to carefully design scenarios to protect students’ emotions [39]. More specific advice for psychological safety is given in research concerning the different sequences of SBE. Psychological safety was found to be established when students experienced that facilitators had an attitude of being friendly, cared about students, were humorous and established a non-competitive environment [40]. It was also found especially challenging for facilitators to re-establish psychological safety in debriefings when students had experienced threats and failures in the scenario. It is recommended that facilitators must be able first to recognize students who signal uncertainties and then restore psychological safety by conveying positive affect and validating and normalizing the student’s frustrations [46]. It has also been identified that facilitators find it challenging to give students clear feedback without harming them. However, this was addressed by seeing the students as resilient and trusting in their ability to tackle critique. This helped facilitators normalize the fact that failure in simulation was common and even desirable to create learning opportunities [47].

However, in a learning situation, psychological safety entails more than removing anxiety-inducing factors in the educational environment. Optimal learning situations should challenge students. Successfully eliciting student engagement, attention and cognition can improve the quality of learning [15, 16, 48]. Triggering emotions such as interest, confusion and curiosity is essential in students’ learning processes because such emotions motivate exploration. Further, students can experience psychological safety and still feel challenged as long as trust and accountability remain [49]. Learning situations where comprehensive emotions are provoked have been found to improve academic achievement [8].

Research and guidelines recommend beneficial facilitator performance in SBE. Students’ anxiety in SBE and facilitators’ responsibility for establishing psychologically safe learning environments are recognized; however, little is known about facilitators’ strategies when handling students’ affect. In teacher and facilitator education, research in the affective learning domain is sparing, despite many learning activities such as SBE eliciting students’ comprehensive or destructive emotions [8]. Studies call for research about how faculty could adapt simulation to learners’ emotions [50].

## Method

### Study aim

This study explores how experienced facilitators approach students’ emotions in SBE and what strategies they use to meet students’ emotional needs in SBE.

Strategy is hereby defined as the plan and actions that facilitators describe to achieve the best learning result during SBE.

### Study design

This qualitative study was guided by interpretive description (ID) (Thorne, 2016). ID is a methodological approach inspired by phenomenology, ethnography and grounded theory and is appropriate when exploring people’s experiences in the applied world. ID acknowledges that researchers enter the research field with various practical backgrounds and experiences which can give valuable insight into the research process [51]. The authors of the present paper had experience teaching and facilitating SBE with nursing and medical students, which provided the team with knowledge about the core challenges in the SBE field. This knowledge permeated the research process. Thorne (2016) states that researchers with experience from the practical field know the contextual background, and that this offers greater credibility and can generate qualitative findings that may be important to practitioners. The authors’ knowledge and experience with SBE were beneficial when discussing challenges on an equal level with the facilitators.

### Ethical considerations

The study was approved by the Norwegian Data Protection Service (ID number 59059). Participants received verbal and written information about the study. Participation was voluntary, and each facilitator signed an informed consent form accepting audiotaping of the interview and permitting interview transcripts to be shared within the research group. Audiotapes were stored in a secured data system to which only the four researchers had access. Data were de-identified when transcribed.

### Participants and data collection

Nine facilitators were strategically recruited from six Norwegian universities. We contacted facilitators from different universities to strive for variation in the participants’ experiences. The first author (AM) contacted the subjects via e-mail. The facilitators who were invited to participate had described their interest in simulation on the University’s web page or their role as facilitators was commonly known in the simulation field. The inquiry inclusion criteria stated that our aim was to contact facilitators who actively operated as facilitators in nursing bachelor or nursing continuing education. Nine of the eleven facilitators we contacted accepted the invitation.

Data collection started in February 2020 and finished in December 2020. Three interviews took place at the facilitators’ workplace, while six interviews were conducted digitally because of Covid-19 pandemic restrictions.

We conducted semi-structured individual interviews. The researcher team prepared the interview guide. The main topics in the interviews were facilitators’ experience of students’ emotions, and their strategies according to students’ emotions when planning and running simulations. Each interview lasted from 54 to 88 min. Interviews were audio-recorded and later transcribed verbatim by the first author.

### Data analysis

The analysis followed the recommendations described in ID [51]. The first step was immersion in the transcripts by listening and reading interviews multiple times. Next, interviews were coded in themes. Themes within each interview were conducted by systemizing statements that shared common opinions. Facilitators’ statements and opinions guided our descriptions and interpretations. In the next step, themes within each interview were compared and patterns were identified. In the final step, the themes were compared and contrasted between the interviews. Interpretation of data is fundamental to ID methodology. Through mulling, interpretive manoeuvre and intellectual interpretations the themes and sub-themes were established.

After each interview, transcription took place with a concurrent analysis. We were then able to gain a deeper understanding and to ask more specific questions during the following interview. All researchers analysed data individually, and authors met regularly to align codes and discuss the ongoing analyses.

## Results

The study aimed to explore how facilitators approached students’ emotions during SBE. The participant sample reflected diversity in gender, educational facilitator background and experiences. Two men and seven women were included. Participants had on average 5,5 years’ experience with facilitating (ranging from two 2–8 years). One participant had no formal training as a facilitator, while the other eight had attended various national facilitator courses offered by universities and commercial enterprises. Two participants used high-technology simulators and six facilitators simulated used low-technology simulation where students portrayed the patient, and one facilitator used a combination of the two approaches.

Table 1, which shows an overview of facilitators strategies related to the affective learning domain in SBE, is presented according to the themes and sub-themes identified in the data analysis. Further, the results are described and exemplified using quotes from the transcripts. The analysis show that facilitator strategies balance between establishing and maintaining psychological safety and evoking emotions to optimize learning in SBE.

**Table 1:** Facilitator’s strategy according to affective learning domain in SBE presented in themes and sub-themes

**Themes**	**Identifying and observing students’ emotions**	**Emotional safeguarding**	**Emotional eliciting**
**Sub-themes**	Observe students’ body language	Prepare by organization	Trigger engagement and interest
	Acknowledge students’ verbally expressing emotions		
	Observe students’ readiness	Adapted knowledge level	Induce surprise
		Reduce students’ anxiety	Encourage learning from mistakes
		Moderate students’ self-criticism	

### Identifying and observing students’ emotions

Facilitators expressed that they were sensitive to students’ emotional reactions in SBE. Anxiety and uncertainty were the most frequently recognized emotions, and these were identified when students verbally expressed their nervousness or when observing students’ body language. For example, when facilitators claimed that students felt uncertain, they based this on their observations of students’ lack of eye contact, and their constant search for support from others in the scenario. Nervous students were experienced performing in a disorganized fashion by being unsystematic, repeating questions and failing to listen or respond to information. According to the facilitators, students’ anxiety was related to students entering an unknown situation and their feeling pressured to perform, and their fear of being revealed as not good enough in front of peers and the facilitator. Facilitators noticed that students often compared their own performance with that of their peers, and that students felt discouraged when they observed gaps between their own knowledge and the knowledge of more confident students. One facilitator expressed this as “*when someone appears to be very good, the other feels stupid”* (F1). Facilitators also claimed that students’ insecurity and nervousness often resulted from a lack of control over the situation. As an example, uncertainty was often observed during the scenario when students verbally expressed that they experienced practical dilemmas in prioritizing tasks.

Embarrassment typically surfaced during debriefing when students discovered their own knowledge gaps or had made mistakes. After SBE was completed, facilitators noted students’ enjoyment when they had felt they had managed the scenario successfully.

### Emotional safeguarding

Facilitators highlighted the time they spent organizing simulations. When the facilitators were well informed about reading lists, curriculum and expected psychomotor skills, and the students’ SBE experience and clinical experience, they could create scenarios adapted to the student’s knowledge level. Adapting to the appropriate student competency level was considered important to avoid students’ frustration and to create manageable scenarios. One facilitator reported *“I focus on creating a safe learning environment by regulating the use of technological opportunities, reducing surprises and narrowing the student’s attention by setting fewer learning goals. I understood that we had to simplify the reality and make the scenarios less complex.”* (F9). To achieve this, facilitators prepared by ensuring that realistic equipment was available and familiarizing with the equipment and manikins before the students entered the SBE. Also, before simulation day, facilitators ensured that the equipment worked as they had intended.

Facilitators were aware of their role as authority figures in the students’ academic lives and tried to minimize their authority status before entering simulations. Some facilitators spent time with the students before the simulation to get to know them and to establish a friendly tone. Several facilitators described how they toned down their teacher role as experts by emphasizing in the prebriefing that they met as equals in the SBE situation.

We found that facilitators strove to reduce anxiety and nervousness by de-dramatizing the learning situation in the prebriefing. They clarified that the simulation was not a test situation and that no one would be judged on their performance. Facilitators were also aware of their behaviour and strove to be calm, clear and non-threatening. As a way to reduce the gap between themselves and the students, and to normalize nervousness, facilitators shared their own simulation experiences and revealed their own failures.

Facilitators urged students to familiarize themselves with the learning objectives to make SBE predictable. Confidentiality in the group was emphasized. Further, they highlighted that in SBE, it is fine to make mistakes. Roles were often assigned ad hoc during the prebriefing, and anxious students were often given less demanding tasks. In addition to verbal guidance facilitators used nonverbal communication such as nodding, smiling, maintaining eye contact and using clear body language to make the students feel safe.

During the debriefing, facilitators helped students avoid being overly self-critical if they performed poorly. Allowing students the opportunity to express their experiences of personal emotions early in the debriefing was crucial because later in the debriefing the students were then able to reflect without focusing on personal emotional experiences. Several facilitators expressed that they addressed students’ emotions early in the debriefing to be able to focus on more professional reflections.

### Emotional eliciting

Facilitators planned scenarios with the intention of stimulating the students, both physically and emotionally. Further, they discussed the importance of realism in scenarios to trigger engagement and interest. Facilitators agreed that realism and the use of realistic equipment were important making the scenario trustworthy. When students experienced realism, they became more interested and engaged in the scenario.

Emotions such as confusion and uncertainty were perceived to enhance learning. Using a (simulated) psychiatric patient to invade the student’s personal space is an example of the use of a scenario plot to trigger a student’s uncertainty. Facilitators also described how they evoked students’ emotions in scenarios by instructing the patients’ relatives to act in demanding, angry or threatening ways. When creating such triggers, facilitators attempted to provoke students’ reflections. These generated discomforts and strong emotional experiences opened for reflections about ethics, responsibility and students’ professional performance. However, facilitators experienced that students became frustrated and irritated if the scenarios became too chaotic. Finding the right balance therefore was deemed important.

To minimize confusion, facilitators highlighted the importance of reducing complexity in the scenario and focusing on just a few expected learning outcomes. One facilitator described adaptation through experience: “*Thinking about what I have changed the most since I started as a facilitator is the change from focusing on creating a high level of realism and surprises to creating a safe learning environment by regulating the use of technological opportunities, reducing surprises and focusing on fewer learning objectives.*” (F9). By simplifying the complexity of realistic patient situations, facilitators experienced an increase in student attention, which helped students to focus on learning objectives.

When facilitators observed student discomfort or unsatisfactory knowledge levels, they adjusted the complexity of the scenario to minimize confusion and frustration. One facilitator explained her method of regulation: *“There are some who quite obviously have a little more control, then I can be more challenging and put on more extra things. I may start to ask*, ‘*how; does that medicine work? to trigger them.”* (F7). One facilitator expressed how the opposite strategy was used when students seemed uncomfortable: *“I do not make crazy situations when I observe uncomfortable students, then I make the simulation less challenging.”* (F4).

Surprise was perceived as a powerful strategy to maintain student attention and interest. Facilitators purposely included content designed to evoke emotions in the scenario. For example, one facilitator explained: “*They know that this patient will get an anaphylactic reaction during the scenario, but they do not know when. So they constantly observe the patient, searching for anaphylactic signals and are in an alert situation.*” (F8). Surprises were increasingly implemented as students gained more experience throughout the educational programme.

Most facilitators indicated that making mistakes during a scenario often optimized the learning process. One facilitator expressed: *“I think that if they have failed on something once, they will remember it for the rest of their lives. They will never forget to switch on that machine again.”* (F7). Facilitators were aware of their power to expose the students when they pointed out their failures, knowledge gaps and poor performance during debriefing. One facilitator said: “*You can really crush them by saying that this was such a poor performance.”* (F1). Facilitators talked about how they strove to protect students who had performed poorly and at the same time felt responsible for educating them according to professional standards. One facilitator expressed this dilemma: *“Simulation is supervision, but when we have to point out failures, we also evaluate their performance.”* (F5). Further, some facilitators expressed that confronting the students with their knowledge gaps prevented the discussion from becoming superficial. Facilitators were aware that such confrontations could sometimes be uncomfortable for students. If students had failed in a scenario, facilitators experienced that providing guidance was challenging. They feared that students’ self-critique could cause shame and that their failures could overshadow the facilitator’s attempt to provide feedback on positive aspects of the students’ performance: *“They only remember the negative things they did.”* (F1)*.* A strategy the facilitators used to handle mistakes was to focus on good and poor performances separately. When addressing poor performances, they would apply an analytic as opposed to an evaluative approach. *“I prefer to focus on small parts and go systematically through her observations. I perform gentle exposure and illuminate one thing a time.”* (F9). By slowly letting the students discover their own failures facilitators tried to reduce humiliation.

To summarize, the findings show that facilitators are aware of students’ emotions during SBE and have numerous strategies for using emotions as catalysts to optimize student learning.

## Discussion

### Facilitators as balancing artists

Our findings show that facilitators are aware of students’ various and individual emotional reactions during SBE and that they have numerous strategies for using emotions to optimize student learning. The strategies include attempts to reduce emotions that might hinder learning, but also efforts to trigger emotions that enhance learning, such as interest, curiosity and surprise. Notably, the strategies were more nuanced than just reducing negative emotions and cultivating positive emotions. In fact, significant learning was seen as something that might also arise from uncomfortable experiences, such as students realizing their own mistakes or uncertainty. We found that facilitators elicited students’ uncertainty and confusion, because such experiences led to reflections and discussions about decision making. A previous study linking confusion to curiosity and engagement supports this, stating that the cognitive disequilibrium students experience during problem-solving resulted in efforts to resolve the discrepancy experienced [15]. However, there is a fine line between productive confusion and unproductive confusion. Too much confusion can lead to frustration and anger and turn focus away from the learning task. On the other hand, confusion can lead to boredom if students give up on solving the problem [15]. Therefore, being awareness of students’ emotional reactions when eliciting confusion is important for assessing each students optimal learning zone.

Similar balancing acts were found concerning eliciting surprise. Facilitators in this study provoked surprise only when students were assesses as academically and psychological ready for such challenge. Previous studies have shown that surprise can benefit learning by increasing cognition, directing attention and promoting curiosity [48]. Solli et al. (2020) found that students who experienced unexpected moments in SBE showed improved learning. In contrast, Monterio and Sibbald (2020) discuss the use of surprise during SBE and argue that empirical evidence does not support the integration of surprises in scenarios. They argue that surprises can induce stress and anxiety, which can impair learning, and that simulation is a naturally complex learning situation that does not benefit from adding additional surprises [52]. Furthermore, surprises in SBE can lead to decreased focus on the planned learning objectives, unless the strategy is well planned and aligned with the learning objectives [53]. Therefore, surprise should be used with caution and preferably in SBE involving experienced students and facilitators [54].

Overall, the key to finding the right balance between safeguarding and eliciting emotions was by observing students’ needs and readiness to engage in SBE. We found that facilitators protected students who did not show readiness and strove to reduce negative emotions such as anxiety because it was perceived to block learning. The observations of students’ reactions helped them identify anxious students who needed reduced complexity and students who were ready for further challenges. Evidence suggests that optimal learning in SBE requires adaptation to individual needs [55, 56]. Our findings showing how facilitators constantly regulate difficulties and challenges to avoid student frustration and thus adapting SBE to students’ individual reactions, are supported in the literature [2, 21, 57]. It is important to remember that students differ significantly in expectations, resilience, previous experiences and level of knowledge, and they will therefore respond differently to challenging situations.

The establishment of psychological safety was found to be an essential requirement to evoke students’ emotions. Facilitators in our study were aware of their responsibilities to ensure inclusiveness, support, trustworthiness and openness. They also had strategies for establishing and maintaining such learning conditions. Psychological safety is highlighted in SBE guidelines, which describe the skills facilitators need to succeed [1]. However, guidelines and recommendations often fail to operationalize ideal facilitation. This study offers insight into the advanced observations, assessments and adjustments that facilitators make during all phases of SBE, with a particular focus on the strategies used to cater to students’ emotions as a part of learning. We were impressed by how many strategies facilitators used and the complex assessments they made to balance group dynamics, thus reducing and eliciting appropriate emotions according to individual and contextual factors. This adds to the existing literature describing the complexity of the facilitator role.

Although few studies in simulation settings have investigated facilitators’ strategies to address students’ emotions during SBE, our findings echo research focusing on affect as an important domain in student learning [8, 11]. SBE has been found to be an emotional endeavour for students and failing to recognize the affective domain can result in suboptimal learning or can block learning [17, 21, 58]. According to the results in this study creating optimal learning situations in SBE involve more than establishing and maintaining a psychological safe learning environment. Facilitators desired to convey surprise, interest, attention and activity to assist the nursing students.

### Relevance for practice

The findings of this study have implications for facilitators of SBE by highlighting the importance of catering to students’ emotions as part of learning. The facilitators participating in this study were attentive, and had a wide repertoire of strategies for being mindful of students’ emotions. Given the complexity of this task, the affective component of learning should be recognized in facilitator training. Facilitators should seek to become aware of which emotional pedagogical strategies they use; for instance, by self-reflection or peer discussions. Furthermore, our findings show that psychological safety is a prerequisite for successful SBE. We therefore suggest that emotional stimuli must co-exist with the establishment and maintenance of psychological safety and be performed when facilitators are in a position to observe and adapt their teaching practices to students’ reactions.

### Limitations

This study showed that facilitators had numerous strategies for safeguarding and eliciting students’ emotions in SBE, yet it is important to highlight that not all participating facilitators used all the strategies. This study did not discuss whether students recognized these strategies, nor if the strategies had the intended effects on student learning. This is an aera of enquiry where further research is warranted, particularly studies exploring how different strategies effect learning outcomes. Some strategies might be more useful than others, but this was beyond the scope of this work. Furthermore, the data are based on the facilitators’ self-reports of intensions and practices which may or may not correspond with their actual behaviours. No objective observations of teacher performance or behaviour were made, leaving facilitators’ ways of practicing SBE open for further exploration.

## Conclusion

This study described and discussed experienced facilitators’ strategies of using emotions to optimize learning in SBE. Facilitators were aware of students’ emotions and had strategies to evoke and protect students’ emotional reactions in SBE. The research findings provide a new insight into the specific strategies facilitators use to cater to students’ emotions in SBE, showing that emotions should be recognized as important domains for eliciting learning. Furthermore, this research can contribute to developing a rationale for making instructional decisions about students’ emotions that can benefit their learning.

## Data Availability

Data are in the form of 9 transcribed interviews. These are confidential and not accessible to the reader. De-identified data are however available from the authors upon reasonable request. Requests to access datafiles in Norwegian should be made in writing to lead author AM.

## Abbreviations

PEARLS: Promoting Excellence and Reflective Learning in Simulation
SBE: Simulation-based education
